# New Drugs, Appliances, and Things Medical

**Published:** 1891-09-05

**Authors:** 


### NEW DRUGS, APPLIANCES, AND THINGS MEDICAL.

[All preparations, appliances, novelties, etc., of which a notice is desired, should be sent for The Editor, to care of The Manager, 140, Strand, London, W.C.]

### AT THE BRITISH MEDICAL ASSOCIATION.

SECTION A.—(*continued*).

An extremely interesting and varied display was made by JOHN RICHARDSON AND CO., of Leicester. Besides the ordinary pharmaceutical preparations, there were very complete exhibits of surgical appliances and medicine cases, and dispensing cabinets. Their well-known coated pills, of course, made a feature in the show. We were particularly pleased with the various methods of medication by means of suppositories, bougies, and pessaries. The firm is always to the fore with any speciality that may prove useful. Here was the new hypnotic "Mikozone," which contains cannabis indica, and henbane with bromide, in a fluid menstruum; "Wytchylene," containing the active principle of the Witch Hazel; the Concentrated Liquors for extemporaneous preparation of syrups, &c.; the etherial tinctures and liniments which Sir J. Sawyer advocates; and the Codeia and Glycerine Jelly of Dr. J. S. Mahomed, all point to the fact that the firm is on the *qui vive* to produce any new form of medication which proves itself of interest to the profession.

JOHNSON AND JOHNSON (LIMITED), Australian Avenue, E.C., occupied the other end of the table with a fine display of plasters, surgical dressings, and Upjohn's pills. Amongst the plasters we noticed especially the belladonna and the emplastrum cantharidis. The first is made from the root of the plant, as the manufacturers state that it is of more constant strength. The blister plaster called "Canthos" will raise a bleb in two or three hours. This is because the excipient contains tar as a foundation. The "Linton" absorbent dressings are sent out ready for use, but in an always moist condition; this is a decided improvement, as the part required for a dressing has merely to be cut off and applied immediately. There is no preliminary wringing out in an antiseptic fluid. The moisture is retained by means of neat tin boxes, in which the dressings are sent out. The Upjohn Friable Pill is a remarkable triumph of invention and manufacturing skill. The drugs are made up into pill form without excipient, and in the state of powder. Coated with sugar, except the reducing agents, which are coated with kaolin. They are practically powders in pill form, and it is claimed for them that they produce the full effect of the drug, which is sometimes diminished by the action, mechanical or otherwise, of the pill excipient with which they are put up. We should like to know the method of manufacture, as how the powder gets in the pill puzzles us, as the presence of the apple in the dumpling did George III. Any formula, we believe, can be made up. The firm already make more than 400 different kinds of the pills.

Next we come to the "Frame Food Extract." We some time back gave a note on this manufacture as being a remarkably successful attempt to produce the salts and extractives, in a concentrated form, from wheat. The extract is the soluble nutritious matter of wheat-bran freed from the husk. It contains over 10 per cent. of wheat phosphates. It is a dry powder free from chemicals. It is in fact, an extract of wheat which may be compared with an extract of beef. It can, however, be used more extensively, as it mixes well with bread, cornflower, cocoa, sweetmeats, &c. The company also showed a "Frame Food" porridge, which is a delicate cooked food, made from wheaten flour, and having its food value increased by the addition of the extract. The extract is also combined with a fruit jelly, and with a drink syrup, both of which seemed to be excellent and tasty productions.

Messrs. Whitaker and Donisthorpe, 12, Oat Lane, London, E.C., exhibited a new dressing material, "Sedox." It is a silky fibre prepared from an Indian plant, capable of absorbing fifteen times its own weight of fluid, and to contain a natural antiseptic, which obviates in many cases the addition of other forms of germicides. The firm is about to make gauze and bandages from it.

(*To be continued.*)

### ANTISEPTIC INDIA-RUBBER CUSHIONS FOR BED PAN, BED SLIPPER, AND BED BATHS.

Messrs. Maw and Thompson have supplied a want that has been long felt in the nursing world. The inadequacy of china bed pans and baths in long illnesses where such are required is well recognised. They are far too hard for patients rendered tender and feeble by ill-health, and the matter of supplying them properly warmed is always a difficulty. This new invention overcomes the difficulty, as the articles are supplied with india-rubber cushion-rims. These rims are hollow, and admit of being filled with warm water on one or both sides of the pan or bath, as is found to be most convenient. The arrangement is not at all cumbersome, and the additional height is very slight. They are perfectly antiseptic, and admit of being thoroughly cleaned and carbolised without difficulty.

